# Tele-cognitive rehabilitation for adult lower-grade glioma: An interim prospective pilot feasibility study

**DOI:** 10.1093/nop/npaf073

**Published:** 2025-07-29

**Authors:** Christina Weyer-Jamora, Melissa S Brie, Paige M Bracci, Ellen M Smith, Tracy L Luks, Stephanie Phan, Steve E Braunstein, Nancy Ann Oberheim-Bush, Nicholas A Butowski, Jennifer L Clarke, Karin Gehring, Adrian Aguilera, John de Groot, Susan M Chang, Shawn L Hervey-Jumper, Jennie W Taylor

**Affiliations:** Department of Psychiatry, Zuckerberg San Francisco General Hospital, San Francisco, California, USA; Department of Neurological Surgery, University of California San Francisco, San Francisco, California, USA; Department of Psychiatry, Zuckerberg San Francisco General Hospital, San Francisco, California, USA; Department of Neurological Surgery, University of California San Francisco, San Francisco, California, USA; Department of Epidemiology and Biostatistics, University of California San Francisco, San Francisco, California, USA; Department of Neurological Surgery, University of California San Francisco, San Francisco, California, USA; Department of Radiology and Biomedical Imaging, University of California San Francisco, San Francisco, California, USA; Department of Neurological Surgery, University of California San Francisco, San Francisco, California, USA; Department of Radiation Oncology, University of California San Francisco, San Francisco, California, USA; Department of Neurology, University of California San Francisco, San Francisco, California, USA; Department of Neurological Surgery, University of California San Francisco, San Francisco, California, USA; Department of Neurological Surgery, University of California San Francisco, San Francisco, California, USA; Department of Neurology, University of California San Francisco, San Francisco, California, USA; Department of Neurological Surgery, University of California San Francisco, San Francisco, California, USA; Department of Cognitive Neuropsychology, Tilburg University, Tilburg, Netherlands; Department of Neurosurgery, Elisabeth-TweeSteden Hospital, Tilburg, Netherlands; Department of Neurological Surgery, University of California San Francisco, San Francisco, California, USA; Department of Neurological Surgery, University of California San Francisco, San Francisco, California, USA; Department of Neurology, University of California San Francisco, San Francisco, California, USA; Department of Neurology, University of California San Francisco, San Francisco, California, USA; Department of Neurological Surgery, University of California San Francisco, San Francisco, California, USA

**Keywords:** brain tumor, cognitive rehabilitation, neuropsychology, telehealth

## Abstract

**Background:**

Cognitive impairments are common in lower-grade gliomas (grades 1–3), but treatment options are limited. Tele-cognitive rehabilitation offers a potential solution. We conducted an interim pilot study to assess the feasibility, satisfaction, and early efficacy of tele-cognitive rehabilitation.

**Methods:**

We enrolled adults with stable LrGG (≥6 months posttreatment) who had subjective and objective cognitive impairments (>1 SD below-average in ≥2 domains). Participants received 3 months of individual Goal Management Training (GMT), app-based ReMind, or texting. Cognition and patient-reported outcomes were assessed at baseline (T1), postintervention (T2), and 9 months postbaseline (T3). We assessed enrollment, adherence, and satisfaction. Adherence was defined as ≥80% of participants completing ≥80% of the protocol; satisfaction as ≥6/7 for GMT and texting, and ≥4/5 for ReMind on a self-report Likert question. We used ANOVA, reliable change indices, and qualitative analytics.

**Results:**

Thirty-nine participants were eligible and 33 prospectively enrolled for the study; an 85% enrollment (17-GMT, 8-ReMind, 8-texting; 46.8 median age, 64.8 months from diagnosis, 55% had astrocytoma, and 76% had prior radiotherapy). Eighty-two percent of GMT (adequate), 100% of texting (adequate), and for ReMind 33% of retraining and 50% of compensation (inadequate) completed ≥80% of the protocol. GMT (mean: 6.75/7) and ReMind (mean: 4.5/5) satisfaction were adequate, and texting (mean: 4.5/7) was inadequate. Working memory improved from T1-to-T2 (*P* = .02, *η*² = 0.32) in 26% of the GMT group.

**Conclusions:**

GMT demonstrates adequate feasibility, satisfaction, and may yield improvements in working memory, while texting and ReMind had challenges in acceptability or feasibility. Individual (tele) GMT warrants further investigation in LrGG.

**Key Points**

- Individual Goal Management Training (GMT) is feasible and shows promise for boosting working memory in low-grade glioma.
- In terms of satisfaction with tele-cognitive rehabilitation approaches, individual GMT and app-based ReMind had adequate satisfaction, while texting interventions had inadequate satisfaction in low-grade glioma.

**Importance of the Study**

This prospective study is the first to evaluate the feasibility of various (tele) cognitive rehabilitation strategies for adults with lower-grade gliomas (LrGG). Our findings contribute to the literature in providing evidence for the feasibility of individual Goal Management Training (GMT). Adherence rates were higher for tele-GMT (93%) compared to in-person (83%), underscoring the potential of remote intervention for individual GMT. Participants valued GMT and ReMind specific strategies, while most texting participants noted dissatisfaction with messages not being more personalized to their symptoms and lacking feedback about their progress. Also, the participants reported technological challenges using ReMind which highlights the importance of screening for digital literacy when using app-based interventions.

Our results align with broader evidence that telehealth approaches are feasible with potential benefit to LrGG survivors. While sample sizes were small, and technical challenges emerged, this study contributes to our understanding of tele-cognitive rehabilitation.

Individuals with primary brain-based lower-grade diffuse gliomas (LrGGs)—grades 2 and 3—have a median survival time of 5 to 15 years^1–3^ depending on the molecular subtype.^4^ However, these tumors and their related treatments often lead to significant objective and subjective cognitive impairments^5–7^ that negatively affect one’s quality of life (QOL).^8–10^ Moreover, cognitive impairments in brain tumor have been associated with higher healthcare utilization^11^ and loss of productivity, including at work.^12^

Cognitive impairments are among the most frequently reported challenges for both patients with brain tumors and their caregivers,^13^ and patients cite treatment for cognitive deficits as a priority in their care.^14^ Still, access to cognitive rehabilitation in brain tumor populations remains limited despite high rates of persistent cognitive complaints. This is thought to be due in part to a limited understanding of the usefulness in brain tumor populations, scarcity of highly trained clinicians, and resource intensity related to in-person intervention.^15^

Cognitive rehabilitation is defined as a systematically applied therapeutic service designed to improve cognitive function and participation in daily activities.^16^ Holistic cognitive rehabilitation programs have existed for 3 decades,^17^ although cognitive rehabilitation interventions were formally noted in the literature after World War I.^18^ The therapeutic mechanism of modern cognitive rehabilitation is based on experience-based neuroplasticity^19^ and neural reorganization crucial for recovery and functional improvements after damage to the brain.

In practice, cognitive rehabilitation often includes *psychoeducation* (cognitive and behavioral) along with a combination of 2 major approaches: (1) *cognitive retraining* that strengthens cognitive functions or specific skills through graded practice and (2) *compensatory strategy training* of applying behavioral strategies to overcome cognitive difficulties and optimize daily functioning.^20^

Goal Management Training (GMT)^21^ is a manualized cognitive rehabilitation intervention for executive functioning for use in both groups and one-on-one/individually. It provides psychoeducation and compensatory strategy training and it has been validated in other neurological populations with promising results in patients with glioma.^22^ GMT aims to increase awareness and understanding of one’s executive dysfunction, provides structured exercises to address cognitive problems, and is gaining evidence in improving aspects of executive functioning.^21^ Meta-analysis of GMT^23^ across 19 studies in populations with mixed neurological (nontumor) etiologies demonstrated beneficial improvements in executive functioning with small to medium effects. GMT in a randomized controlled study by Richard and colleagues (2019) of low- and high-grade glioma, meningioma, and other brain tumor types 9 years post-initial antitumor treatment showed promising results. Mean adherence (*n* = 11) to GMT was remarkably high (99%) with noted improvement in executive function at 4 month follow-up. Additionally, patient QOL factors such as self-reported cognitive complaints improved while emotional coping remained unchanged after GMT.^22^ That said, the feasibility and efficacy of telehealth deployed GMT in LrGG populations remains unclear.

Cognitive rehabilitation such as GMT in glioma may show promise, although evidence remains sparse with limited availability. Therefore, evidence for accessible interventions leveraging technology to address cognitive impairments remains needed, albeit currently is at a nascent stage. Van der Linden et al. investigated^24,25^ the cognitive rehabilitation app (ReMind) in patients with low-grade glioma and meningioma 3 months after surgery. ReMind is a remotely guided, self-paced app-based cognitive rehabilitation program providing compensation and attention retraining through psychoeducation and exercises targeting attention, memory, and planning.^25^ Van der Linden included patients with low-grade glioma and meningioma 3 months after surgery.^24,25^ On average, participants completed 85% of compensation and 91% of attentional retraining components. Also, 90% of participants rated ReMind as good or excellent. Performance-based cognitive and patient-reported outcomes were observed to improve more after ReMind (*n* = 23) than in the control group (*n* = 26) but not significantly, although statistical power was limited.

Short messaging services (SMS) or text is another cost-effective technological approach to providing psychoeducation. In texting, individuals receive nonpersonalized psychoeducational text messages to help them learn information and strategies to manage disease-related symptoms, without the need to reply. Texting interventions have promising results such as adjunctive treatments for patient education, appointment reminders, and interventions for disease states such as depression (Healthy SMS), smoking cessation, and diabetes control. However, little is known about their use in cognitive rehabilitation for individuals with brain tumors.^26,27^

We hypothesized that individuals with LrGG would (1) be interested in one-on-one, app-based, or psychoeducational text messages; (2) recruitment, enrollment, and adherence would be adequate; and (4) participants would report satisfaction with the intervention. Secondarily, for groups greater than 10 we wanted to explore improvements in cognition after treatment.

## Methods

### Study Participants

Participants were recruited from the UCSF Division of Neuro-Oncology during their routine follow-up appointments. Criteria for eligibility were: (1) ≥ 18 years; (2) confirmed diagnosis of radiologically stable lower-grade (grades1, 2, and 3) supratentorial primary glioma as defined by the WHO 2016 criteria^28^; (3) KPS ≥ 70; (4) English fluency for speaking and reading; (5) internet and phone access; (6) clinically stability and off tumor treatment (craniotomy, radiation, or chemotherapy) for ≥ 6 months; (8) self-reported cognitive impairment complaints to their neuro-oncologist; (9) adequate seizure control; (10) ≥ 1 SD below their estimated premorbid level of cognitive functioning on ≥ 2 domains of baseline cognitive assessments (Table 1). Impairments in executive functioning were not required for eligibility. The Barona Index^29^ and Wechsler Test of Adult Reading^30^ (WTAR) measured premorbid intellectual functioning to estimate cognitive change compared to estimated pretumor functioning.^31^

**Table 1.** Cognitive Assessments

Cognitive test	Functional areas	Scores used	Norms Reference
**Premorbid Intellectual**			
WTAR	Word reading	Total score	Wechsler 2001^30^
Barona	Demographically derived	Total score	Barona, 1984^29^
**Attention and Processing Speed**			
WAIS-IV Working Memory Index (WMI; Digit Span and Arithmetic subtests)	Auditory working memory	Total score	Wechsler, 2008^32^
TMT-A^a^	Visuomotor sequencing and psychomotor speed	Total time	Heaton et al., 2004^33^
SDMT (oral)	Mental processing speed	Total score	Smith, 1982^34^
**Memory and Learning**			
HVLT-R with parallel forms	Verbal memory and learning	Trails 1-3 total recall, delayed recall	Benedict and Brandt, 2007^35^
BVMT-R with parallel forms	Visuospatial memory and learning	Trails 1-3 total recall, delayed recall	Benedict and Brandt, 2007^35^
**Language**			
NAB—screening object naming	Object naming	Total score	Stern and White, 2003^36^
Animal naming	Semantic generation of animals	Total score	Heaton et al., 2004^33^
**Executive Functioning**			
COWAT	Response generation of letters FAS	Total score	Heaton et al., 2004^33^
TMT-B^a^	Set shifting and cognitive flexibility	Total time	Heaton et al., 2004^33^
TOL^a^	Planning and organization	Total score	Culbertson and Zillmer, 2005^37^
DKEFS Color Word Test^b^	Set shifting and inhibition	Total score	Delis et al., 2001^38^

Wechsler Test of Adult Reading (WTAR); Wechsler Adult Intelligence Scale (WAIS-IV) Working Memory Index-Digit Span and Arithmetic subtests; Trail Making Test-A (TMT-A); Symbol Digit Modalities Test (SDMT) oral version; Hopkins Verbal Learning Test (HVLT): Brief Visuospatial Memory Test (BVMT); Neuropsychological Assessment Battery (NAB) Naming Screening subtest; Trail Making Test-B (TMT-B); Tower of London (TOL); Controlled Oral Word Association Test (COWAT); and Delis Kaplan Executive Function Scale Color Word Subtest.

^a^Tests not administered once transitioning to remote testing on March 2020.

^b^Tests added after the transition to remote testing.

Participants were excluded if they were (1) diagnosed with glioblastoma or grade 4 IDH mutated tumors as defined by WHO 2016; (2) determined by a licensed neuropsychologist to be unable to participate in cognitive testing due to the severity of deficits; or (3) acutely psychiatrically distressed; active psychosis, suicidality, and/or were psychiatrically gravely disabled.

The UCSF institutional review board approved the study protocol (NCT03948490). All procedures were performed following ethical standards of the Helsinki Declaration and participants provided written informed consent.

### Study Design

**Figure 1.**
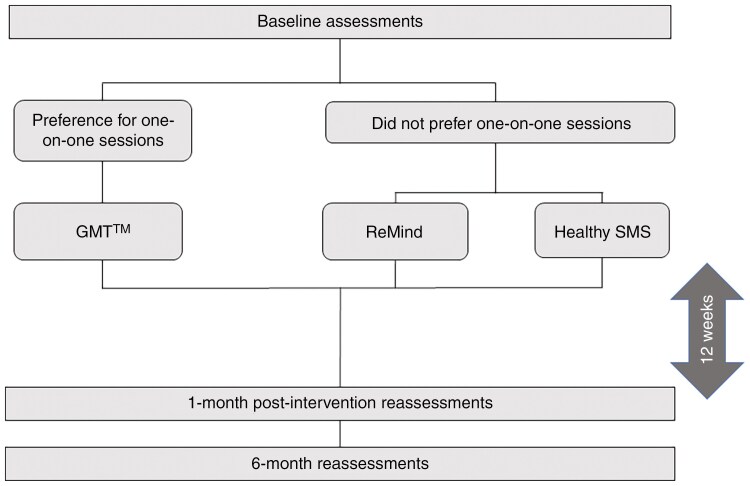
Study Design. Eligible participants were first given the option to attend one-on-one sessions. Participants who agreed to participate in one-on-one sessions went on to receive GMT. If participants declined to participate in one-on-one sessions, they were randomized 1:1 to ReMind or Healthy SMS. All participants received 12 weeks of their prescribed intervention, followed by cognitive reassessment 1 month postintervention and again 6 months postintervention.

Participants were invited for consent within 1 month of their recent MRI and neuro-oncology appointment between 2019 and 2022. Consented participants completed cognitive testing, and self-reported satisfaction measures. Eligible participants were first offered (1) one-on-one cognitive rehabilitation with (individual) GMT and were instructed if they declined GMT they could be randomized to either (2) remotely guided app-based cognitive rehabilitation (ReMind) or (3) psychoeducational text messaging (Healthy SMS) (Figure 1).

Of note, our study was launched approximately 9 months before the COVID-19 pandemic. Upon opening the study, our intention was to study the feasibility of offering in-person GMT, versus lower touch options such as app-based and text messaging. We initially chose this methodology because many patients travel long distances to attend appointments and randomization into in-person options would be unduly burdensome. Therefore, our study design offered participants in-person rehabilitation or randomization to texting or app-based intervention. However, COVID disrupted these plans necessitating GMT be deployed via telehealth. Altering our study to be fully randomized was considered but discarded due to internal validity concerns. Therefore, after March 2020, GMT was converted to video-based telehealth visits (tele-GMT), with participants still being first offered one-on-one tele-GMT and then, if they declined, were randomized to ReMind or Healthy SMS.

### Interventions/Study Arms

Eligible participants completed 3 months of one intervention (GMT, ReMind, or Healthy SMS). Cognition was reassessed within 1 month postintervention, and 6 months postintervention (Figure 1).

[1] *GMT*^21^: Nine manualized modules were adapted to 6 interactive 60–75-min sessions delivered bi-weekly over 3 months by a licensed psychologist with postdoctoral training in rehabilitation neuropsychology and trained in GMT. GMT was deployed in an individual rather than group format to give more individual attention to the application of GMT concepts and provide a more private, confidential space for exploration of presented topics. Exercises were designed to illustrate and improve goal-directed behavior with homework assignments designed to facilitate transfer of concepts to real life (Supplementary Table 1). As specified by GMT, participants tracked and discussed cognitive symptoms in session to facilitate GMT principles application to their symptoms. To guide individual GMT and later telehealth deployment, authors engaged GMT publishers (Baycrest) for consultation regarding one-on-one and telehealth use and restructuring multiple modules to be given per session. Modifications to study materials such as workbooks were mailed to participants (telehealth), and more than one module was administered per session (Supplementary Table 1) were noted. Other study materials such as the instructional materials via Power Point slides as recommended by Baycrest remained unchanged.
[2] *ReMind*^24^: App-based cognitive rehabilitation ReMind was provided via iPad by study staff as previously described by Gehring and colleagues. Participants were advised to work with ReMind 3 hours weekly over 3 months. ReMind included compensation training with psychoeducation, and attention retraining components; both are self-paced and can switch between compensation and retraining. Six compensation modules included strategy training for attention, memory, and planning with embedded exercises to help participants learn and practice applying strategies to their daily lives. For attentional retraining (C-Car), participants were encouraged to complete three to four 30-min sessions weekly, completing hierarchically graded exercises designed to target 4 different modes of attention (sustained, selective, alternating, and divided attention) in a driving videogame-based format. Participants initially met remotely with research staff for instruction and were given a written schedule of the order of the assigned modules and retraining for completion. Research staff called participants by phone every 2 weeks to check in on their progress and offered to troubleshoot technical issues (Supplementary Table 2).
[3] *Healthy SMS Texting*^26^: Education via text message was provided regarding common symptoms and strategies for management. Text messages focused on psychoeducation related to health-related QOL and cognitive education such as organizational tips, fatigue management, and coping skills (Supplementary Table 3). Texts were developed at UCSF by an interdisciplinary team including a neuro-oncologist, neuropsychologist, and health psychologist. The texts addressed 4 areas: 1) cognition (27 messages, example “Keep all important objects in an assigned place”); 2) fatigue/sleep (19 messages, example “Limit caffeine after 2 pm since it can make it harder to sleep at night”); 3) mood (23 messages, example “Sticking to a routine can help you feel calmer and less overwhelmed”); and 4) general guidance (20 messages, example “Learning to manage your symptoms can take time and practice. If something doesn’t work, try a different strategy”). Participants received a daily message Monday through Friday sent at a random time within their chosen timeframe(s) (eg, 9:00 am–9:00 pm) for 3 months, and participants were not required to respond to texts.

### Study Assessments

Recruitment, enrollment, intervention assignment, attrition/retention, satisfaction, and delivery consistency were assessed. Also, signal change to detect potential cognitive improvements were assessed in groups with sample sizes >10.

Number of participants recruited, intervention assignment, and attrition and retention were tracked with reasons for ineligibility and attrition recorded. Deviations from delivery consistency were tracked (for GMT administering modules out of sequence or missing modules, for ReMind app instability preventing completion of assigned modules or verifying completion, and for Healthy SMS text platform instability negatively impacting texts being deployed). Interventional satisfaction was determined based on participant responses on posttreatment satisfaction surveys within 1 month after intervention completion.

#### Cognitive testing.

—Assessments were completed at baseline, within 1 month postintervention, and 6 months postintervention (Figure 1) and administered by study staff who were trained by a licensed psychologist with a specialization in rehabilitation neuropsychology. Cognitive functioning was assessed for domains of (1) attention and processing speed; (2) memory and learning; (3) language; and (4) executive functioning (Table 1). Parallel forms were given to minimize practice effects.

Prior to March 2020 all testing sessions and GMT interventions were in-person and after March 2020 all assessments and GMT were administered remotely (see Table 1). Prior to March 2020 cognitive testing included: Wechsler Test of Adult Reading (WTAR), Barona, WAIS-IV working memory index (WMI) digit span and arithmetic subtests,^32^ Trail making test,^33^ Symbol Digit Modalities (oral),^34^ Hopkins Verbal Learning Test (HVLT-R),^36^ Brief Visuospatial Memory Test (BVMT-R), Neuropsychological Assessment Battery (NAB)-screening object naming,^36^ Controlled Oral Word Association Test (COWAT),^33^ and Tower of London (TOL)^37^ were administered in-person. After March 2020, the remote battery consisted of WTAR, Barona, WAIS-IV WMI digit span and arithmetic subtests, Symbol Digit Modalities (oral), HVLT-R, BVMT-R, NAB-screening object naming, COWAT, and DKEFS Color Word^38^; essentially Trail making and TOL were dropped and DKEFS added. Remote tests were administered in accord with the InterOrganizational Practice Committee Recommendations/Guidance for Teleneuropsychology (TeleNP) in Response to the COVID-19 Pandemic. Raw test scores were normatively corrected for demographic factors such as age, sex, and education per scoring rubrics and transformed to *z*-scores.

#### Patient satisfaction.

—Participants were given posttreatment satisfaction surveys within 1 month after the conclusion of their intervention. The GMT and texting group satisfaction survey questions were on a 7-point Likert scale that ranged from 1-strongly disagree to 7 strongly agree. Example questions included “I was happy with …overall” and “The service added value to my care,” as well as an open-ended question (for example “Additional comments/suggestions”). Quantitative satisfaction was determined based on the question of being overall happy with the intervention (Supplementary Table 8). For ReMind, postintervention surveys from the developers were used with satisfaction survey questions on a 5-point Likert scale ranging from 1-poor to 5-excellent. Example questions included “What did you think overall of using this program for learning how to manage your cognitive difficulties?” and “What did you think of the ease of using this program for learning how to manage your cognitive difficulties?” as well as an open-ended question, for example, “Additional comments/suggestions.” For ReMind, responses to the question asking about overall satisfaction using ReMind to manage cognitive difficulties was used to determine the satisfaction with the intervention. Qualitative satisfaction was derived from the free text comments in the respective satisfaction surveys.

Clinical data such as tumor type (by WHO 2016), medications, and treatment history prior to baseline testing were extracted from medical records.

### Data Analysis

Demographic and clinical characteristics were calculated for the total group and statistically compared (with Pearson Chi-square Tests and Spearman Tests) for the 3 interventional groups. Statistical comparison (with ANOVA *F*-tests, Kruskal–Wallis Tests), and reliable change indices (RCIs) of cognitive test scores from pre- to postintervention were used to detect signal change within groups for sample sizes greater than 10.

Enrollment rate sufficiency was not defined prior to the study. Retention was deemed sufficient if there was <20% dropout in each cohort arm after initiating treatment. Adherence was adequate if 80% of the cohort participants completed ≥ 80% of the assigned intervention. For GMT 80% of the sample must complete ≥80% of the assigned intervention including attending (either in-clinic or video visit) ≥5 sessions and assigned modules; for ReMind 80% of the sample must complete ≥80% of each of the 2 assigned components (attentional retraining and compensation training) and completion must be verified by research staff upon return of the study iPad; and for Healthy SMS 80% of the sample must receive ≥80% of assigned texts without opting out. Satisfaction was determined based on quantitative and qualitative feedback via a posttreatment survey at the end of each intervention.^38,39^ For quantitative analysis, descriptive statistics of responses to closed-ended questions in the posttreatment surveys were generated. Sufficient satisfaction was quantitatively defined as a Likert scaling of ≥6/7 for GMT and HealthySMS, and ≥4/5 for ReMind on the aforementioned overall satisfaction survey questions. Qualitatively, responses to an open-ended satisfaction responses were coded and analyzed using inductive thematic analysis^39,40^ which involved independent reading and coding of keywords, phrases, and concepts by 2 team members (CWJ and MB). Disagreements in coding were negotiated until consensus was reached.^41^ Descriptive statistics were used to analyze and visualize coded concepts.

Preliminary efficacy was assessed using demographically corrected *z*-scores from cognitive assessments for within-group repeated measures analysis of variance (ANOVA) comparing baseline to 1-month posttreatment for intervention cohorts with a sample size >10. Eta^2^ effect sizes were computed as the sum of squares of the predictor divided by the total variance (small effect 0.01–0.06, medium effect 0.06–0.14, and large effect ≥0.14). For nonnormal distributions, the Wilcoxon signed-rank test was used to evaluate the significant within-group differences and *r*-effect estimates. Effect sizes were classified as small effect 0.0–0.3, medium 0.3–0.5, and large effect ≥0.50.^42^ Given the primary outcome of this pilot study was feasibility and the preliminary nature of our analyses, no corrections for multiple statistical comparisons were made. To account for practice effects on significant pre–post findings, RCIs were calculated on cognitive scores with significant (*P* < .05) ANOVA and Kruskal–Wallis Test findings at baseline and within 1 month postintervention (T1 to T2). RCIs were calculated by comparing the change in individual cognitive scores to observed changes in published test–retest data. Reliable improvement was defined as RCI values above +1.645 and decline below −1.645 (based on an alpha of 0.10, corresponding to a 90% CI).^43^ RCIs were calculated over the first time interval (baseline-1 month postintervention). Numbers of participants who reliably improved/declined were aggregated and proportionally compared.

## Results

### Recruitment, Withdrawal, and Randomization

**Figure 2.**
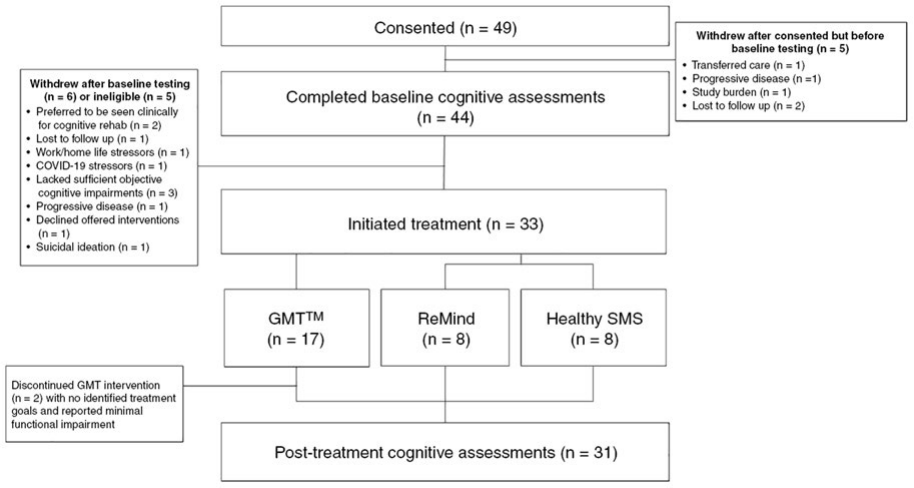
Study Flow Diagram. Abbreviation: GMT, individual goal management training.

Between May 2019 and March 2022, 49 participants provided consent. Five withdrew before baseline testing (1 transferred care, 1 had progressive disease, 1 due to study burden, and 2 were lost to follow-up). After consenting, 5 participants were considered ineligible for the following reasons: 3 people lacked sufficient objective cognitive impairments; 1 person had progressive disease; and 1 person reported suicidal ideation. Another 6 withdrew posttesting (Figure 2) and they noted reasons for withdrawal after baseline testing included: preferred to be seen clinically for cognitive rehabilitation (*n* = 2), lost to follow-up (1), declined offered interventions (1); work and home life stressors (1), and COVID-19-related stressors (1). Thirty-nine met study eligibility including neuropsychological impairment of which 33 agreed to be enrolled in the study; an 85% enrollment rate.

About half of the (consenting and eligible) participants (17/33) preferred GMT (8 in clinic, 9 video visits). Eight were randomized to ReMind; and 8 to Healthy SMS Texting (Figure 2). Prior to the COVID pandemic, 40% who initiated treatment opted for GMT, while after/during the pandemic, 69% opted for GMT (Supplementary Table 4).

### Participant Characteristics

Participants were primarily female (67%), and Caucasian (76%), had a mean of 17 years of education (SD = 2.8), and a mean age of 47 years (SD = 10.9) at the time of enrollment. Most participants were diagnosed with astrocytoma (55%) or oligodendroglioma (30%), WHO grade 2 (55%) and predominantly frontal lobe (70%) lesions. Most participants had prior radiotherapy (76%) and the median time from the initial diagnosis was 65 months (Table 2). After the first 8 GMT participants, the intervention was converted to video visits due to COVID-19 restrictions. As there were no significant demographic and cognitive differences between these groups (Supplementary Tables 6 and 7), all GMT participants were combined into one group for analysis (Table 3).

**Table 2.** Demographic and Clinical Characteristics of 33 Patients with Lower-Grade Glioma (LrGG) by Cognitive Rehabilitation Intervention

	Cognitive Rehabilitation Groups				*P*-value
	GMT (*N* = 17)	ReMind (*N* = 8)	Healthy SMS (*N* = 8)	Total (*N* = 33)	
Age, years (mean, SD)	46.5 (13.9)	48.6 (9.3)	45.4 (9.3)	46.8 (10.9)	.780^a^
Education, years (mean, SD)	16.9 (1.2)	16.4 (3.5)	17.6 (4.3)	17.0 (2.8)	.469^a^
Female gender (*n*, %)	9 (53%)	6 (75%)	7 (88%)	22 (67%)	.197^b^
Tumor pathology (*n*, %)					.451^b^
Astrocytoma	10 (59%)	3 (37.5%)	5 (62.5%)	18 (55%)	
Oligodendroglioma	6 (35%)	3 (37.5%)	1 (12.5%)	10 (30%)	
Other^c^	1 (6%)	2 (25%)	2 (25%)	5 (15%)	
Tumor Grade (*n*, %)					.158^b^
Grade 2	7 (42%)	5 (63%)	6 (75%)	18 (55%)	
Grade 3	10 (58%)	2 (25%)	2 (14%)	14 (42%)	
Grade 1	0	1 (12%)	0	1 (3%)	
Ethnicity (*n*, %)^c^					.778^b^
Caucasian	12 (71%)	7 (87%)	6 (75%)	25 (76%)	
Latino	2 (12%)	1 (13%)	1 (12.5%)	3 (9%)	
Asian-American	2 (12%)	0 (0%)	0 (0%)	2 (6%)	
Other	1 (5%)	0 (0%)	0 (0%)	3 (9%)	
Working at time of enrollment (*n*, %)^c^	9 (53%)	4 (50%)	4 (50%)	17 (52%)	.950^b^
Tumor location (*n*, %)					.241^b^
Frontal	10 (59%)	5 (62.5%)	8 (100%)	23 (70%)	
Insular	0 (0%)	1 (12.5%)	0 (0%)	1 (3%)	
Parietal	4 (24%)	1 (12.5%)	0 (0%)	5 (15%)	
Temporal	3 (17%)	1 (12.5%)	0 (0%)	4 (12%)	
Prior Chemotherapy (*n*,%)	14 (87.5%)	4 (57.1%)	7 (87.5%)	25 (81%)	.202^a^
Prior Radiotherapy (*n*, %)	13 (76%)	5 (63%)	7 (88%)	25 (76%)	.504^b^
Months from last radiation treatment (median, IQR)	51.7 (113.0)	29.7 (12.3)	40.3 (22.1)	39.0 (47)	.390^a^
Months from initial diagnosis (median, IQR)	64.8 (125.6)	102.6 (155.6)	55.6 (138.2)	64.8 (127.9)	.530^a^

Tele-GMT: Goal Management Training via telehealth. Time since initial diagnosis is defined as months since initial surgery.

^a^Pearson chi-square test.

^b^Kruskal–Wallis Rank Sum Test.

^c^Missing: 3 patients had unknown working status and 1 patient had unknown ethnicity.

**Table 3.** Preintervention Neuropsychological Z-scores for 33 Patients with Lower-Grade Glioma (LrGG) by Cognitive Rehabilitation Intervention

	Cognitive Rehabilitation Cohorts								
	GMT		ReMind		Healthy SMS		Total		*P*-value
	Mean (SD)	*N*	Mean (SD)	*N*	Mean (SD)	*N*	Mean (SD)	*N*	
**Premorbid intellect (mean, SD)**									
WTAR	0.66 (0.90)	16	0.52 (1.28)	8	0.94 (0.48)	8	0.69 (0.91)	32	.866^a^
Barona	0.95 (0.32)	17	0.87 (0.28)	8	0.79 (0.34)	8	0.89 (0.31)	33	.497^b^
**Attention and processing speed (mean, SD)**									
SDMT	−1.92 (0.79)	16	−2.07 (0.87)	8	−2.26 (1.41)	7	−2.04 (0.95)	31	.748^b^
TMT-A	−1.26 (1.00)	15	−1.55 (1.38)	8	−2.23 (1.15)	8	−1.58 (1.18)	31	.175^b^
WAIS IV-WMI	−0.38 (1.04)	17	−0.20 (1.05)	8	−0.58 (1.30)	7	−0.38 (1.08)	31	.790^b^
**Memory and learning (mean, SD)**									
HVLT-R 1-3	−0.83 (0.98)	17	−1.60 (0.79)	8	−1.25 (1.24)	8	−1.12 (1.03)	33	.202^b^
HVLT Delay	−0.95 (1.27)	17	−1.83 (1.06)	8	−1.28 (1.40)	8	−1.24 (1.27)	33	.283^b^
BVMT-R 1-3	−0.59 (1.12)	16	−0.21 (1.32)	8	−0.11 (1.67)	8	−0.38 (1.30)	32	.649^b^
BVMT-R Delay	−0.36 (1.43)	16	−0.15 (1.65)	8	−0.05 (1.59)	8	−0.23 (1.48)	32	.881^b^
**Language (mean, SD)**									
NAB Naming	0.35 (1.05)	17	0.29 (1.34)	8	0.44 (0.05)	8	0.36 (0.97)	33	.325^a^
Animal Naming	−0.22 (1.12)	17	−0.68 (0.81)	8	−0.98 (1.19)	8	−0.52 (1.09)	33	.251^b^
**Executive Functioning (mean, SD)**									
TOL	−0.15 (1.14)	15	−0.12 (0.73)	8	−0.21 (1.03)	8	−0.17 (0.99)	31	.992^b^
TMT-B	−1.14 (1.15)	15	−1.30 (1.57)	8	−1.96 (1.32)	8	−1.39 (1.31)	31	.360^b^
COWAT	−0.68 (1.18)	17	−0.83 (1.04)	8	−1.54 (0.82)	8	−0.92 (1.10)	33	.186^b^
FrSBe	0.98 (1.47)	17	1.83 (0.74)	8	1.99 (0.94)	8	1.43 (1.27)	33	.099^a^

^a^ANOVA *F*-test.

^b^Kruskal–Wallis Test.

### Attrition, Deviations in Delivery, and Adherence

Despite initially meeting eligibility criteria of self-reporting cognitive impairments, 2 participants discontinued GMT after a few sessions secondary to reporting their cognitive issues not being functionally impairing enough to warrant intervention. No participants stopped the other interventions. Thirty-one (94%) were reassessed at 1 month postintervention and 23 (70%) at 6-month follow-up (Figure 2). No deviations from GMT protocol were reported.

For adherence, 82% (14/17) of the GMT group completed ≥80% of the assigned intervention (adequate); 8 in-clinic GMT participants and 9 tele-GMT participants. Of the in-clinic GMT group 83% completed ≥80% of the assigned intervention whereas 93% for tele-GMT completed ≥80% of the protocol.

Two out of 8 ReMind participants experienced app instability issues. They reported completing the program, but this could not be verified due to technical issues and thus were excluded from the adherence analysis.

For ReMind, 33% (2/6) completed at least 80% of the assigned attentional retraining. 50% (3/6) completed at least 80% of compensation training. One patient reported their limited technological abilities impacted their ability to use ReMind and demonstrated the lowest adherence.

All (8/8) of the Healthy SMS (adequate) received ≥80% (ie, 100%) of the text messages; no participants opted out. No deviations from delivery were reported.

### Participant Intervention Satisfaction

**Figure 3.**
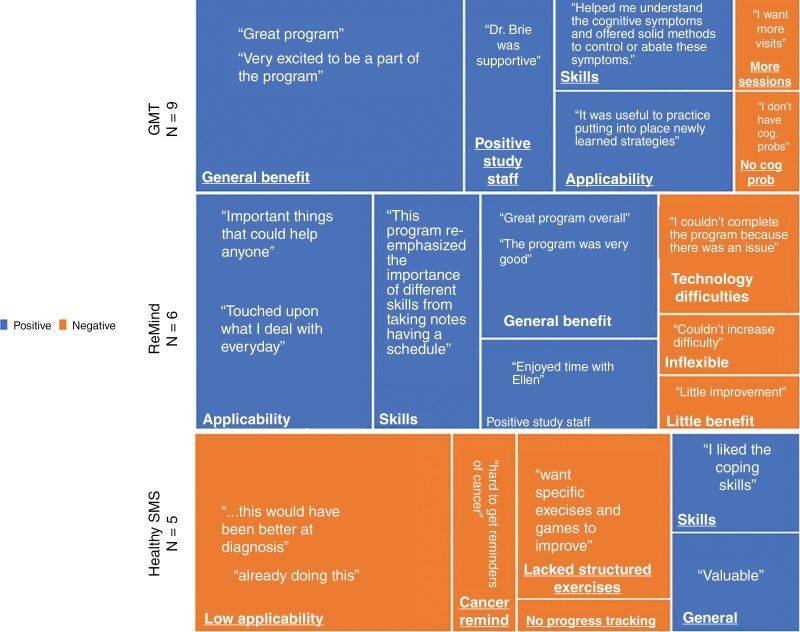
Tree Map of Participant Feedback Question “Additional Comments/Suggestions.” The identified key themes were synthesized from participant quotes and depicted below. Please note participants had experiences in more than one category. Blue boxes indicate positive feedback. Orange boxes indicate negative feedback. The size of each box represents the frequency of the category.

Of the 31 participants who completed the interventions, 68% (ten on GMT; 6 on ReMind; and 5 on Healthy SMS) responded to a posttreatment feedback survey about their experience with the intervention. One GMT participant provided contradictory qualitative and quantitative feedback, suggesting a mis-keying error and one GMT participant did not respond to the overall satisfaction question (although they responded to the other satisfaction questions and provided additional comments). Both could not be included in the quantitative analysis. For qualitative analysis, 9 GMT, 6 ReMind, and 5 Healthy SMS texting people responded with additional comments (Figure 3).

#### GMT.

—Quantitatively, (100%) of the 8 GMT participants were satisfied overall (67% or 6/8 endorsed “strongly agree” and 2/8 endorsed “agree”, mean = 6.75 out of 7) 100% strongly agreed (6/9) or agreed (3/9) that the service added value to their care (mean = 6.67 out of 7). Those seen pre-COVID had a GMT satisfaction of 6.8/7 and post-COVID had a GMT satisfaction of 6.6/7. Assessment of qualitative feedback noted 19 positive sentences and 2 negative sentences. Key themes of the “positive” feedback including (1) general benefits; “Overall this experience was great,” (2) positive interactions with study staff; “Dr. Brie was supportive.,” (3) skills learned; “Helped me to understand the cognitive symptoms and offered solid methods to control or abate these symptoms” and (4) applicability; “It was useful to practice putting into place newly learned strategies.” Negative GMT feedback included a desire for more sessions to work on skills and another person felt GMT was not a fit for them because they felt they did not have significant cognitive problems. One person had mixed (positive and negative) comments whereby she felt the intervention was helpful but felt overwhelmed with implementing the content at the end and wanted more sessions to master the material.

#### ReMind.

—100% (6/6) participants were overall satisfied and (3 endorsed “Excellent” and 3 endorsed “Good” with a mean 4.5 out of 5). And, while 83% (5/6) felt the compensation exercises were useful, 33% (2/6) reported technical frustrations; one related to their learning the app and app stability and the other reported frustration with the game not being customizable to their preferred difficulty level. In the ReMind group, 83% (5/6) said the training they received to use the app by the research staff was satisfactory and 50% (3/6) said they used the learned strategies in their daily lives.

Assessment of qualitative feedback noted fifteen positive sentences and 3 negative sentences. Key themes of the “positive” feedback included: (1) General benefit; “Great program overall,” (2) Skills; “This program re-emphasized the importance of different skills from taking notes and having a schedule,” (3) Applicability; “Touched upon what I deal with everyday,” and (4) positive interactions with study staff “I enjoyed the time I spent with Ellen.” Negative feedback included participants wanting more control of advancing the difficulty level on the retraining component, more time to complete the components, and app instability. There was one mixed response that the participant liked the program overall but felt the “driving [ie, the game] exercises would have been more valuable to me if I was able to increase the difficulty.”

#### HealthySMS texting.

—Forty percent (2/5) reported overall satisfaction (1/5 “agree,” 1/5 “strongly agree” with a mean score of 4.8 out of 7) with the texts, and 20% (1/5) reported it added value to their care. Assessment of qualitative feedback noted 3 positive sentences and 6 negative sentences. Key themes of the “positive” feedback included: (1) General benefit; “The messages were a good reminder of overall health and well-being,” and (2) Skills; “It was good especially with reminders of how to cope with stress.” Negative feedback included feeling the messages did not “apply to me because I was already doing most of the recommendations daily,” wanting messages to be personalized to their symptoms, not wanting daily reminders of their cancer, wanting more feedback and tracking of their progress after using strategies, and more specific and interactive exercises to help them address their symptoms. HealthySMS texting had no mixed responses.

### Preliminary Efficacy for GMT

Analysis of cognitive *z*-scores using parametric statistics for fifteen GMT participants (2 lacked postintervention testing) demonstrated statistically significant improvement from baseline to 1 month postintervention on WAIS-IV WMI with medium effect (*F*-statistic = 5.73, eta2 = 0.32, p = 0.02) (Supplementary Figure 1). No other statistically significant cognitive changes were noted. Computed RCI found 26% of the GMT group had reliable WMI improvement from baseline to within 1 month posttreatment with 0% reliably declined functioning. Due to small sample sizes, cognitive outcomes for ReMind and Healthy SMS were not statistically analyzed.

## Discussion

This is the first prospective study evaluating feasibility of several (tele) cognitive rehabilitation strategies in adult patients with LrGG. Our study found that individual GMT-based cognitive rehabilitation holds promise due in part to the strong feasibility and satisfaction seen in our sample. App-based ReMind demonstrated adequate satisfaction, while Healthy SMS texting was able to be deployed with few opting out.

Overall, adherence to GMT was adequate, and higher for those who received the intervention via telehealth. ReMind did not meet feasibility thresholds for this study due in part to technological issues. For example, the 2 lastly included participants experienced app instability and one participant showed limited ability to use the app’s technology (the latter of whom demonstrated the lowest adherence). According to developers, ReMind is currently undergoing redevelopment to address app stability. Additionally, future studies with app-based platforms such as ReMind may consider screening target populations for digital (il)literacy when considering computerized interventions, and/or provide more direct guidance. As the Healthy SMS intervention consisted of receiving text messages, adherence cannot be well interpreted, although no one opted out of receiving the messages.

This study found GMT and ReMind had the highest satisfaction and met cutoffs for sufficient satisfaction while Healthy SMS texting did not. GMT and ReMind participants remarked enjoying learning specific skills to manage their symptoms, support for putting interventions in place, and the interventions were relevant to their challenges. Healthy SMS’s lower satisfaction was due in part to having discomfort with receiving a daily reminder of their brain cancer diagnosis and struggling to make use of the information on their own. Furthermore, SMS participants described wanting more feedback about their efforts and specific cognitive strategy exercises which were perceived as lacking in the intervention. Our findings align with prior studies supporting structured learning when deploying cognitive interventions.^44,45^ Further, this feedback speaks to the magnitude of impact this diagnosis has on participants’ lives and the vital role clinicians can play in adapting information for use in practice. Rather than a standalone intervention, texting may have potential as an adjuvant treatment for engaging in personalized cognitive rehabilitation programs, such as reinforcing discussed content, reminders about upcoming appointments, and deploying questions to gauge treatment response in cognitive rehabilitation.^26^ Also, patient perspectives were not solicited to inform the text message development of which future research should consider.

For GMT and ReMind, participants reported they liked learning skills that were applicable to their daily functioning, which likely enhanced the satisfaction of these interventions. Qualitative feedback from participants in the GMT group noted a desire for more sessions to master learned skills. This observation aligns with our clinical practice of cognitive rehabilitation at UCSF, in which the average patient engages in approximately 10 sessions of cognitive rehabilitation. Future studies may consider adding visits, as some patients may prefer additional practice and support to consolidate new skills to manage their cognitive symptoms.

Preliminary analysis noted encouraging results for improvements in working memory after GMT with 26% having reliable improvement when accounting for practice effects. Our findings are consistent with a recent meta-analysis of GMT that found improvements in working memory across 8 studies.^46^ Our study adds to the body of literature supporting the use of GMT to address working memory impairments and preliminarily in LrGG survivors, and thus warrants further investigation.

In other populations, Internet-based (neuro)psychological interventions (including self-help and web-based) were demonstrated to have similar or better effect sizes as traditional face-to-face therapies, especially when support is provided.^47,48^ Tele-cognitive rehabilitation increases the availability of cognitive rehabilitation for patients with brain tumors, who often depend on their caregivers for transportation. Web-based interventions (such as ReMind and tele-GMT) allow patients to work on their rehabilitation from the comfort of their home with fewer barriers to access that are common to this population (for example, not being able to drive, and not living near a major medical center). Indeed, the adherence to tele-GMT was higher than when we deployed it in-person. Furthermore, apps have a natural life cycle that can lead to technological instability as happened during this study with ReMind. Issues such as app stability, digital literacy, and related support needs can be helpful to consider when deploying app-based cognitive rehabilitation interventions.

Given these interim results, we amended our protocol to stop enrolling into the ReMind and Healthy SMS cohorts and continued enrollment only into (tele) GMT. Further analysis of a larger GMT cohort outcomes with longer follow-up is forthcoming.

This study has several limitations. First, sample sizes were exceedingly small and an acceptable enrollment rate was not a priori defined. In addition, the participants were first offered GMT and the participant's rationale for interventional selection was not systematically tracked. Offering GMT first may have created an allocation bias with the more motivated patients opting for this intervention. Furthermore, we did not use a control group; therefore, cognitive improvements after GMT need further validation and are not powered for between-group comparisons. Additionally, it is unknown if the posttreatment improvements translated into increased functional independence. Further, our sample is predominantly highly educated and Caucasian; therefore, the generalizability of these findings is limited. Lastly, this study bridged both pre- and post-COVID-19 pandemic including telehealth and in-person GMT. While no significant demographic and cognitive differences between these groups were found, this variability in deployment remains a potential threat to internal validity. Inadvertently, bridging to telehealth during COVID-19 further demonstrated the necessity of these remote interventions.

In conclusion, GMT is feasible and acceptable for adults with LrGG. Additionally, there is preliminary evidence for cognitive improvement after GMT. Future studies should screen for digital literacy, investigate interventions designed to address patients’ concerns, involve clinician support, and highlight applicability to daily functioning.

## Supplementary material

Supplementary material is available online at *Neuro-Oncology Practice* (https://academic.oup.com/nop/).

npaf073_Supplementary_Materials

## Data Availability

The datasets generated during and/or analyzed during the current study are available from the corresponding author upon reasonable request.
