# Supplementary material for: Tele-cognitive rehabilitation for adult lower-grade glioma: An interim prospective pilot feasibility study
Source: Neurooncol Pract. 2025 Jul 29;13(1):126–38. doi: 10.1093/nop/npaf073 (PMC12965652; doi:10.1093/nop/npaf073)
Supplement: npaf073_Supplementary_Materials [file npaf073_supplementary_materials.docx]

**Supplemental Table 1.** Goal Management Training intervention components

| Module | Session number | Description |
| --- | --- | --- |
| 1 | 1 | Focuses on introduction to goal hierarchies and concepts of absent-mindedness and present mindedness with participants completing mindful meditation and symptom monitoring worksheets. |
| 2 | 1 | Reviews the relationship between absent-mindedness to other abilities, consequences of slips, and how GMT reduces slips with participants completing mindful meditation, clapping tasks, and expanded absent-mindedness monitoring worksheets. |
| 3 | 2 | Focuses on how lack of present focus (i.e. automatic pilot) leads to errors in daily life and diminished goal-directed activity with participants completing homework such as expanded symptom monitoring worksheets and mindful meditation. |
| 4 | 2 | Focuses on specific behavioral techniques to stop the automatic pilot as well as completing in-session exercises to gain mastery with homework of completing symptom monitoring, STOP exercises, and mindful meditation. |
| 5 | 3 | Focuses on helping participants understand the concept of a mental blackboard and practice skills such as stop-focus-check with homework exercises focused on tracking the use of skills taught, mindful meditation, and successes with using the technique. |
| 6 | 4 | Focuses on learning about goal loss and how to reinstate goals using the stop-state cycle. |
| 7 | 5 | Focuses on learning about goal conflict and decision-making with exercises to learn organizational strategies, practicing the stop-state cycle, and related homework to practice these skills between sessions. |
| 8 | 5 | Focuses on learning skills to deal with overwhelming tasks by splitting them into small tasks with practice using organizational strategies and a stop-state-split chart. |
| 9 | 6 | Focuses on reducing impulsiveness by using checking strategies and addresses how to continue to implement learned strategies over time. |

**Supplemental Table 2.** ReMind intervention components

| **ReMind app for Compensation training and Retraining** | |
| --- | --- |
| Compensation training consisting of psychoeducation and teaching of strategies provided in video, audio and text, and multiple fill-in exercises to practice the strategies provided in daily life, and C-Car Attention Retraining Game. | |
| Week 1 Compensation  Module 0  Introduction | Focus on general introduction to the program, compensation training, and attention retraining. |
| Week 2 Compensation  Module 1  Cognitive functions | Focus on cognitive function including: Attention, planning and control, memory, and interconnection of cognitive functions. |
| Week 3 and 4 Compensation  Module 2  Influences | Focus on daily-life factors that influence cognitive functioning include psychological factors and strategies such as relaxation exercises. |
| Week 5 Compensation Module 3  Compensation | Focus on the influence of general conditions on cognition (optimal time, rest, environment, and motivation), applying strategies, and using tools. |
| Week 6 and 7 Compensation  Module 4  Attention | Focus on strategies for aiding selective, sustained, alternating, and divided attention, and strategies for reading text. |
| Week 8 and 9 Compensation  Module 5  Planning & control | Focus on strategies to plan and execute an activity (7-Step Schedule), planning multiple activities in a day, using a calendar, and using an action-focused list. |
| Week 10 and 11  Compensation  Module 6  Memory | Focus on short-term memory, long-term memory, conditions that can impact memory, strategies, and tools. |
| Week 12 Compensation Module 7  Strategy ratings | The patient completes a rating scale regarding the usefulness of the strategies that were provided. |
| Attention Retraining  Three to four 30-minute separate sessions/week | Focus on the completion of hierarchically graded exercises aimed at training sustained, selective, alternating, and divided attention in a driving game format. The exercises have visual and auditory elements with both verbal and numeric stimuli. |
| Additional Features | There are options to send e-mails to their significant other, make personal notes, a definition list for key words used in the program, and personal identification number for privacy. |
| *Note: Patients can complete the entire program largely independently; however, to ensure sufficient adherence, they had remote guidance from study staff who contacted them via phone every two weeks to check on their progress.* | |

**Supplemental Table 3.** HealthySMS text messages

| **Domain** | **Text content** |
| --- | --- |
| **Cognitive**  **Cognitive**  **Fatigue/ Sleep** | \| C01\|Heads Up: Do one thing at a time if it's hard to focus. Notice when your attention is  drifting and gently direct your attention back on task. Rate your concentration today from  1 to 4 (1 = best, 4= worst) \| \| --- \| \| C02\|Heads Up: If you are tired and have trouble sleeping, take a few deep breaths.  Poor sleep can lead to feeling distracted. Take a rest and try to refocus. \| \| C03\|Heads Up: It can be hard to pay attention when trying to multi-task and can lead to  overwhelm. Keep it simple and do one thing at a time. Rate your concentration today from  1 to 4 (1 = best, 4 = worst) \| \| C04\|Heads Up: Write important things down immediately. Use a calendar to keep track of  appointments and information. When in doubt, write it down! \| \| C05\|Heads Up: Keep all important objects in an assigned place (keys, wallet, phone). Make  sure to return things to where they belong every time. \| \| C06\|Heads Up: Try to avoid distractions and noise when concentrating. Clear out visual  clutter. Try headphones to dampen noise if you need to. Rate your concentration today from  1 to 4 (1 = best, 4 = worst) \| \| C07\|Heads Up: When learning new things, rehearse it to get it right. Involve your senses: Say  it out loud, read it, imagine it in your mind, and repeat it back. \| \| C08\|Heads Up: It can be easy to get mixed up when doing things. Set a goal, plan a solution,  and implement it. What worked? What do you need to do differently next time?  Take notes and try again. \| \| C09\|Heads Up: Some people remember visual information easier than words. Try thinking in  pictures! Rate your memory today from 1 to 4 (1 = best, 4 = worst) \| \| C10\|Heads Up: If you can't find a word, try slowing down how fast you are talking. Pause,  and try to describe it instead. \| \| C11\|Heads Up: Create a daily/weekly routine, practice makes perfect! Assign a day of the  week for tasks (such as groceries on Tuesdays, lunch at noon). \| \| C12\|Heads Up: Link behaviors that happen together naturally (such as taking medications  at mealtimes). \| \| C13\|Heads Up: If the word doesn't come to you while you are talking, see if you can think of  something related. Even if it isn't quite right, it may get your point across. \| \| C14\|Heads Up: Get thoughts out of your head and onto paper: Make and review a "to do"  list daily. \| \| C15\|Heads Up: Review your "to do" list daily. If you notice certain tasks keep carrying over,  reflect on why. Re-prioritize, set realistic limits, and ask for help. \| \| C16\|Heads Up: If you are having a hard time making a decision: Try writing down your goal  and related pros and cons. \| \| C17\|Heads Up: Create a single workplace for things that require concentration. It will ensure  that you automatically associate that place with working attentively. \| \| C18\|Heads Up: Use tools that make your life easier and increase self-confidence, such  as calendars, address books, recipes, and shopping lists. \| \| C19\|Heads Up: When you need to concentrate, find a quiet environment. Close the door,  switch off your phone, and use earplugs if necessary. Put everything you need in that room  before you start. \| \| C20\|Heads Up: Planning means being able to set your goal beforehand and knowing which  steps you need to take to reach that goal. Plan ahead (get your bag ready the night before,  review your calendar daily, etc.). \| \| C21\|Heads Up: Making a schedule for different steps of a project will help you to keep an  overview of what you are doing. \| \| C22\|Heads Up: Often, it can be helpful to use tools to support your memory. Think of a  notepad, electronic tools, or objects that help remind you to do something. \| \| C23\|Heads Up: Taking notes can improve your memory! Making notes makes you focus your  attention on specific information, which improves your memory. \| \| C24\|Heads Up: To help your memory, write things down immediately, take a picture, or use  a voice recorder on your phone. When in doubt, make a note. \| \| C25\|Heads Up: Keep things you look for regularly in a fixed and logical place, such as keys or  reading glasses. Make a habit of always leaving them in that place. \| \| C26\|Heads Up: When you realize you are being distracted, try to eliminate the nuisance. If  necessary, use earplugs. \| \| C27\|Heads Up: If you notice it is hard for you to do things for long periods of time, dividing  tasks into smaller steps can help. \| \| F01\|Heads Up: Learning to pace yourself is important. The best way to begin pacing is to be  more aware of your energy level and to plan your activity accordingly. \| \| F02\|Heads Up: Many times, you can start to have more energy just by being more aware of  overdoing/underdoing and planning your day accordingly. Rate your energy level today from  1 to 4 (1 = best, 4 = worst) \| \| F03\|Heads Up: When individuals with brain tumors are overly tired, they will experience  symptom flares such as increased forgetfulness, distractibility, and emotional reactions. Rest  can help! \| \| F04\|Heads Up: Even after having a good night’s rest, you may not feel fully recharged. Take  more breaks and slow down how much you do. Rate your sleep last night from 1 to 4  (1 = best, 4 = worst) \| \| F05\|Heads Up: When you have a brain tumor, your brain has to work much harder to  complete tasks. Pay attention to what helps and focus on what works to recharge. \| \| F06\|Heads Up: Fatigue is very common. Pace yourself! Physical exercise guided by listening  to your body can help. Rate your fatigue today from 1 to 4 (1 = best, 4 = worst) \| \| F07\|Heads Up: Scheduling breaks, whether you need them or not, helps to manage  exhaustion. Have a back-up plan in case you can't get everything done. \| \| F08\|Heads Up: How well did you pace your activities today? Rate your fatigue today from  1 to 4 (1 = best, 4 = worst) \| \| F09\|Heads Up: Overdoing can make fatigue worse, but underdoing can lead to overdoing  later. Listen to your symptoms and set realistic limits based on the energy you have. \| \| F10\|Heads Up: To manage fatigue, figure out what time of day and what environment helps  you do your best (morning, dim light, quiet). Make your environment work for you! \| \| F11\|Heads Up: To manage fatigue, do the things that you need to do first and let other things go. Come back to it later when you are fresher. \| \| F12\|Heads Up: Go to sleep at night at the same time, and limit day naps to 20 minutes if you  are having trouble sleeping at night. \| \| F13\|Heads Up: Have a bedtime routine. Do relaxing things before bedtime, such as a warm  bath/shower. Rate your sleep last night from 1 to 4 (1 = best, 4 = worst) \| \| F14\|Heads Up: Bed is only for sleep and intimacy. No screentime in bed. Don't toss and  turn- get up if you are awake for more than 20 minutes until sleepy again. \| \| F15\|Heads Up: Limit caffeine use (especially after 2 pm). It can make it harder to sleep at  night. \| \| F16\|Heads Up: Being mindful of your energy level can help you learn how much energy you  need for each task. Rate your energy today from 1 to 4 (1 = best, 4 = worst) \| \| F17\|Heads Up: Pay attention to your energy level while doing tasks. Were you surprised with  how much energy it took? What did you learn about what you need to be successful? \| \| F18\|Heads Up: Many people feel more energetic in the morning than in the afternoon or  evening. Think about which part of the day you feel most fresh. You can plan more important  tasks for that time of day. \| \| F19\|Heads Up: An important strategy in preventing fatigue is to stop an activity when you start  to feel tired. Many people with brain tumors notice that they feel delayed fatigue, such  as the day after a busy day. \| \| G01\|Heads Up: Brain tumors can cause changes in thinking, seeing, talking, feelings, and how  you move your body. Symptoms vary depending on the type of tumor, location, and size. \| \| G02\|Heads Up: If you have had surgery, recovery can be smooth, or it can take more time. Be  patient with yourself and take breaks as needed. \| \| G03\|Heads Up: You can be more sensitive to alcohol and drugs, and for some, it can make  symptoms worse. \| \| G04\|Heads Up: Learning to manage your symptoms can take time. If something doesn't work,  try a different strategy. Rate your symptoms today from 1 to 4 (1 = best, 4 = worst) \| \| G05\|Heads Up: Remember, headaches are common with brain tumors. Try to limit any personal habits that lead to worse headaches (skipping meals, stress, overexertion, dehydration, etc.) \| \| G06\|Heads Up: Drinking enough water, reducing smoking, regular exercise, and eating regular  meals can help with headaches. Rate your pain today from 1 to 4 (1 = best, 4 = worst) \| \| G07\|Heads Up: Overusing caffeine and getting too stressed make headaches worse: Try  limiting caffeine and taking 3 deep breaths to relax. \| \| G08\|Heads Up: Attending a support group can be a great way to feel better. UCSF has a  monthly Brain Tumor Support Group. Talk to your doctor to learn more! \| \| G09\|Heads Up: Make sure you have the time to perform a certain task. Plan ahead to do as  much as you can in advance to avoid a time crunch. Choose a moment that fits the kind of  activity in question. \| \| G10\|Heads Up: Try to schedule thoughtfully. If you dislike busy environments, plan visits at a  quiet moment. Do your grocery shopping in the morning, for example, when it is still quiet in  the stores. \| \| G11\|Heads Up: Routines can save you lots of time and effort. Try to make frequently  recurring tasks a habit. \| \| G12\|Heads Up: When you need to do a challenging activity, prepare before you start. Select a  start and end time. Schedule a break to relax, take a walk, or do something that requires less  attention. \| \| G13\|Heads Up: When planning activities, schedule breaks and ending times. If necessary, set  an alarm. Try it and rate your success from 1 to 4 (1 = best, 4 = worst) \| \| G14\|Heads Up: Being disturbed by distracting sounds makes it hard to concentrate. What  distracts you the most? Track what helps you to feel better. \| \| G15\|Heads Up: If you do not have a lot of time, do not start a task that you know will be  difficult for you to stop. Save that task for when you have more time. \| \| G16\|Heads Up: Good schedules require time and discipline, but in the end, they will save you  time. This will give you peace of mind. Try to stick to your schedule and see how it works! \| \| G17\|Heads Up: Activities often take more time than we think. It is wise to schedule more  time for your activities than you think you will need. \| \| G18\|Heads Up: Make priorities. Decide which tasks really need to be done and which ones  can be either skipped or postponed. \| \| G19\|Heads Up: If you make a schedule for your day, evaluate it at the end of the day. Don't  be hard on yourself if you can't stick to the schedule. Think about how you can make future  schedules work better. \| \| G20\|Heads Up: Check your calendar at fixed moments, such as in the morning to see what  you have planned for the day, and in the evening to prepare the schedule for the next day. \| \| M01\|Heads Up: Thoughts at night can keep you awake. Try a relaxation exercise, like  imagining being in a place you find relaxing and take some deep breaths. \| \| M02\|Heads Up: Being hard on yourself can make it even harder to concentrate; slow down  and be kind to yourself. \| \| M03\|Heads Up: You may find it harder to control what you do and feel. Slow down, think  calming thoughts, and take a few deep breaths. \| \| M04\|Heads Up: The more time you spend doing pleasant and healthy activities, the better  you will feel. Go for a walk! Talk to a friend! \| \| M05\|Heads Up: Sticking to a routine can make you feel calmer and less overwhelmed.  Calendar your appointments and plans as you go along. Check your calendar each day. \| \| M06\|Heads Up: For those with brain tumors, irritability, sadness, and nervousness are  common. Talking to your doctor is an important first step to feeling better. \| \| M07\|Heads Up: Your symptoms can make it harder to spend time with others, but loneliness  can make your mood worse. Talk with others to think of some ways you can get the  support you need! \| \| M08\|Heads Up: When you feel sad, doing a small activity can make you feel better. Try doing  an activity, even if you do not feel like it. You may start to feel better after you start. \| \| M09\|Heads Up: Think about your day. Rate your mood today from 1 to 4 (1 = best, 4 = worst).  Can you think of a thought that improved your mood? Keep practicing! \| \| M10\|Heads Up: Unhelpful thoughts make our mood worse. Think about an unhelpful thought  you had today. Can you think of a way to change it into a helpful thought? \| \| M11\|Heads Up: You may get frustrated or impatient when your expectations are not met.  Track your triggers! When this happens think about what you have control over. What  can you let go? \| \| M12\|Heads Up: How is your mood today? Rate your mood today from 1 to 4  (1 = best, 4 = worst) \| \| M13\|Heads Up: Being quick to anger after a brain tumor is common. Try to notice early when  you are upset and take a time out. Come back when you are calmer. \| \| M14\|Heads Up: Stress can slow you down and make you feel exhausted. Rate your stress  today from 1 to 4 (1 = best, 4 = worst). Track your stress triggers and make a self-care plan. \| \| M15\|Heads Up: How stressed did you feel today? Rate your stress today from 1 to 4  (1 = best, 4 = worst) What helps you feel better? \| \| M16\|Heads Up: Feeling worried? Worries can pile up. Try writing down your worries, then  look and see what you can control and what you can let go for now. \| \| M17\|Heads Up: One of the best medicines for your mood is to move your body. Make time  for simple activities that give you pleasure. For example, get outdoors and go for a walk. \| \| M18Heads Up: If you are feeling tense, take a break, and take 3 deep, slow breaths. Rate  your stress today from 1 to 4 (1 = best, 4 = worst) \| \| M19\|Heads Up: When you feel annoyed, remember you don't have to react right away. Take  a break. Try to say, "I'm going to think that over and get back to you." \| \| M20\|Heads Up: Take a break and get into the now: run water on your hands, eat a piece of  fruit, meditate, and drink a cup of tea. What do you hear, smell, taste, touch & see? \| \| M21\|Heads Up: Take time to sit or lie down somewhere quiet and relax your body especially  when you are tired and/or stressed. \| \| M22\|Heads Up: If you tend to be hard on yourself, take a breath and imagine what you  would say to a loved one who was talking that way about themselves. Be kind! \| \| M23\|Heads Up: Try doing things that make you calm, relaxed, and satisfied, you will release  tension. Examples are gardening, taking a bath, taking a walk, or listening to music. \| |
| **Fatigue/ Sleep**  **General** |  |
| **General**  **Mood** |  |
| **Mood** |  |

**Supplemental Table 4.** Patient-by-patient assessment of the feasibility of cognitive rehabilitation interventions

| **Participant** | **Consent** | **Module Type** | **% completion of assigned intervention** | **Reported technical difficulties** |
| --- | --- | --- | --- | --- |
| Participant 1 | Pre-COVID | GMT (in clinic) | 100% | n/a |
| Participant 2 | Pre-COVID | GMT (in clinic) | 100% | n/a |
| Participant 3 | Pre-COVID | GMT (in clinic) | 83% | n/a |
| Participant 4 | Pre-COVID | GMT (in clinic) | 83% | n/a |
| Participant 5 | Pre-COVID | GMT (in clinic) | 66% | n/a |
| Participant 6 | Pre-COVID | GMT (in clinic) | 33% | n/a |
| Participant 7 | Pre-COVID | GMT (in clinic) | 100% | n/a |
| Participant 8 | Pre-COVID | GMT (in clinic) | 100% | n/a |
| Participant 9 | Post-COVID | GMT (video visit) | 100% | No |
| Participant 10 | Post-COVID | GMT (video visit) | 100% | No |
| Participant 11 | Post-COVID | GMT (video visit) | 100% | No |
| Participant 12 | Post-COVID | GMT (video visit) | 33% | No |
| Participant 13 | Post-COVID | GMT (video visit) | 100% | No |
| Participant 14 | Post-COVID | GMT (video visit) | 100% | No |
| Participant 15 | Post-COVID | GMT (video visit) | 100% | No |
| Participant 16 | Post-COVID | GMT (video visit) | 100% | No |
| Participant 17 | Post-COVID | GMT (video visit) | 100% | No |
| Participant 18 | Pre-COVID | ReMind C-Car | 63% | Yes^a^ |
|  |  | ReMind Compensation | 48% |  |
| Participant 19 | Pre-COVID | ReMind C-Car | 100% | No |
|  |  | ReMind Compensation | 100% |  |
| Participant 20 | Pre-COVID | ReMind C-Car | 68% | No |
|  |  | ReMind Compensation | 100% |  |
| Participant 21 | Pre-COVID | ReMind C-Car | 61% | No |
|  |  | ReMind Compensation | 55% |  |
| Participant 22 | Pre-COVID | ReMind C-Car | 68% | No |
|  |  | ReMind Compensation | 73% |  |
| Participant 23 | Post-COVID | ReMind C-Car | 100% | No |
|  |  | ReMind Compensation | 100% |  |
| Participant 24 | Post-COVID | ReMind C-Car | n/a | Yes^b^ |
|  |  | ReMind Compensation | n/a |  |
| Participant 25 | Post-COVID | ReMind C-Car | n/a | Yes^b^ |
|  |  | ReMind Compensation | n/a |  |
| Participant 26 | Pre-COVID | Texting psychoeducation | 100% | No |
| Participant 27 | Pre-COVID | Texting psychoeducation | 100% | No |
| Participant 28 | Pre-COVID | Texting psychoeducation | 100% | No |
| Participant 29 | Pre-COVID | Texting psychoeducation | 100% | No |
| Participant 30 | Pre-COVID | Texting psychoeducation | 100% | No |
| Participant 31 | Pre-COVID | Texting psychoeducation | 100% | No |
| Participant 32 | Pre-COVID | Texting psychoeducation | 100% | No |
| Participant 33 | Post-COVID | Texting psychoeducation | 100% | No |

GMT: Individual Goal Management Training

GMT participant completion was defined as the percentage of completed assigned intervention.

ReMind completion was defined as the percentage of completed assigned interventions.

Texting completion was defined as the percentage of assigned text messages without requesting to opt-out.

N/A signifies that technical difficulties did not apply due to the intervention being deployed in person (i.e. in clinic GMT).

^a^ participant reported technical difficulties due to their own ability

^b^ participant reported app-based technical difficulties/instability of the app and was unable to verify component completion

Pre-COVID (prior to March 2020)

**Supplemental Table 5.** GMT within-group paired comparisons at baseline and post-intervention

|  | GMT | | | |
| --- | --- | --- | --- | --- |
|  | Statistic | Paired N | P value | Effect Size |
| **Attention and processing speed** | | | | |
| SDMT | 0.25 | 13 | 0.62 | 0.21 |
| TMT-A^a^ | 22.00 | 11 | 0.61 | 0.20 |
| WAIS-IV-WMI | 5.73 | 15 | 0.02 | 0.32 |
| **Memory and learning** | | | | |
| HVLT-R Trials 1-3 | 1.94 | 15 | 0.19 | 0.12 |
| HVLT Delay^a^ | 18.00 | 15 | 0.36 | 0.29 |
| BVMT-R Trials 1-3 | 0.60 | 15 | 0.46 | 0.05 |
| BVMT-R Delay | 0.01 | 13 | 0.92 | <0.01 |
| **Language** | | | | |
| NAB Naming^a^ | 7.00 | 15 | 0.58 | 0.25 |
| Animal Naming | 0.08 | 15 | 0.79 | 0.01 |
| **Executive Functioning** | | | | |
| TOL | 0.70 | 11 | 0.42 | 0.07 |
| TMT-B | 0.25 | 11 | 0.63 | 0.03 |
| COWAT | 0.36 | 15 | 0.56 | 0.03 |
| FrSBe | 0.22 | 13 | 0.82 | 0.02 |

GMT: Individual Goal Management Training

Wechsler Adult Intelligence Scale (WAIS-IV) Working Memory Index-Digit Span and Arithmetic subtests; Trail Making Test-A (TMT-A); Symbol Digit Modalities Test (SDMT) oral version; Hopkins Verbal Learning Test (HVLT): Brief Visuospatial Memory Test (BVMT); Neuropsychological Assessment Battery (NAB) Naming Screening subtest; Trail Making Test-B (TMT-B); Tower of London (TOL); Controlled Oral Word Association Test (COWAT); Frontal Systems Behavior Scale (FrSBe).

^a^ Wilcoxon sign rank test for non-normal distributions with r for effect size.

**Supplemental Table 6.** Demographic and clinical characteristics of GMT group pre and post COVID

|  | **GMT Cognitive Rehabilitation Groups** | | **P value** |
| --- | --- | --- | --- |
|  | **Pre COVID**  **(N=8)** | **Post Covid**  **(N=9)** |  |
| Age, years (mean, SD) | 47.5 (10.4) | 49.0 (10.5) | 0.385^a^ |
| Education, years (mean, SD) | 16.0 (1.0) | 16.0 (1.4) | 0.698^a^ |
| Female gender (n, %) | 3 (37.5%) | 6 (66.6%) | 0.229^b^ |
| Tumor pathology (n, %) | | | 0.510^b^ |
| Astrocytoma | 4 (50%) | 6 (67%) |  |
| Oligodendroglioma | 3 (37%) | 3 (33%) |  |
| *Other | 1 (13%) | 0% |  |
| Tumor Grade (n, %) | | | 0.486 ^b^ |
| Grade 2 | 4 (50%) | 3 (33%) |  |
| Grade 3 | 4 (50%) | 6 (67%) |  |
| Ethnicity (n, %) * | | | 0.815 ^b^ |
| Caucasian | 6 (75%) | 6 (67%) |  |
| Asian American | 1 (12.5%) | 1 (11%) |  |
| Latino | 1 (12.5%) | 1 (11%) |  |
| Other | 0% | 1 (11%) |  |
| Tumor location (n, %) |  | | 0.871 ^b^ |
| Frontal | 5 (63%) | 5 (55%) |  |
| Parietal | 2 (25%) | 2 (22.5%) |  |
| Temporal | 1 (12%) | 2 (22.5%) |  |
| Prior Chemotherapy (n,%) | 6 (75%) | 8 (100%) | 0.131 ^b^ |
| Prior Radiotherapy (n, %) | 6 (75%) | 7 (78%) | 0.893 ^b^ |
| Overall satisfaction (n, mean, SD) | 6 (6.8, 0.4) | 2 (6.5, 0.7) | 0.51^c^ |

GMT: Individual Goal Management Training

^a^ T-Test

^b^ Chi Square

^c^ Mann Whitney

**Supplemental Table 7.** Pre-intervention neuropsychological Z-scores for in-person and tele-health deployed GMT

|  | **GMT Cognitive Rehabilitation Cohorts** | | | |  |
| --- | --- | --- | --- | --- | --- |
|  | In-person | | Tele-health | | P value |
|  | Mean  (SD) | N | Mean  (SD) | N |  |
| **Premorbid intellect (mean, SD)** | | | | |  |
| WTAR | 0.76 (0.49) | 8 | 0.55 (1.13) | 8 | 0.60^a^ |
| Barona | 0.93 (0.36) | 8 | 0.95 (0.28) | 9 | 0.70^b^ |
| **Attention and processing speed (mean, SD)** | | | | | |
| SDMT | -1.78 (0.67) | 8 | -2.06 (0.91) | 8 | 0.48 ^a^ |
| WAIS IV-WMI | -0.15 (1.01) | 8 | -0.53 (1.07) | 9 | 0.46 ^a^ |
| **Memory and learning (mean, SD)** | | | | | |
| HVLT-R 1-3 | -0.63 (0.94) | 8 | -1.01 (1.02) | 9 | 0.43 ^a^ |
| HVLT Delay | -0.84 (1.19) | 8 | -1.05 (1.39) | 9 | 0.73 ^a^ |
| BVMT-R 1-3 | -0.28 (1.24) | 8 | -0.90 (0.95) | 8 | 0.28 ^a^ |
| BVMT-R Delay | 0.13 (1.54) | 8 | -0.85 (1.21) | 8 | 0.18 ^a^ |
| **Language (mean, SD)** | | | | | |
| NAB Naming | 0.76 (0.83) | 8 | -0.01 (1.12) | 9 | 0.13 ^a^ |
| Animal Naming | -0.47 (1.07) | 8 | 0.40 (1.18) | 9 | 0.40 ^a^ |
| **Executive Functioning (mean, SD)** | | | | | |
| COWAT | -1.00 (1.01) | 8 | -0.40 (1.29) | 9 | 0.31^a^ |
| FrSBe | 0.91 (1.67) | 8 | 1.04 (1.35) | 9 | 0.86 ^a^ |

GMT: Individual Goal Management Training

^a^ T-test

^b^ Mann-Whitney

**Supplementary Table 8.** GMT satisfaction survey


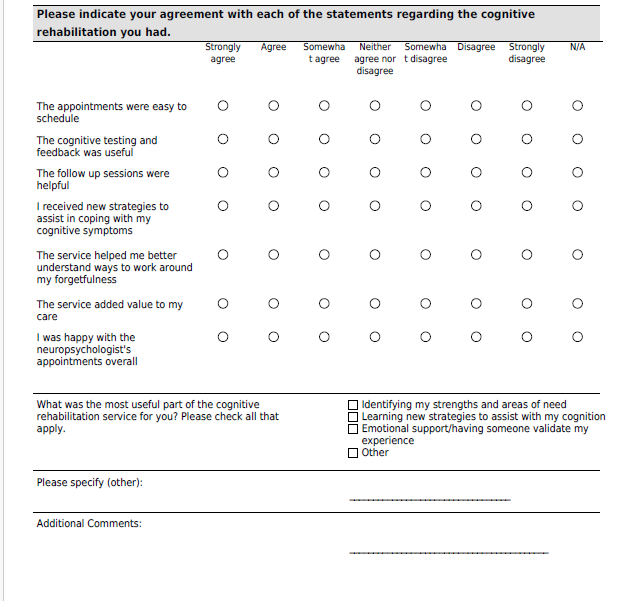


**Supplementary Table 9.** ReMind satisfaction survey

**
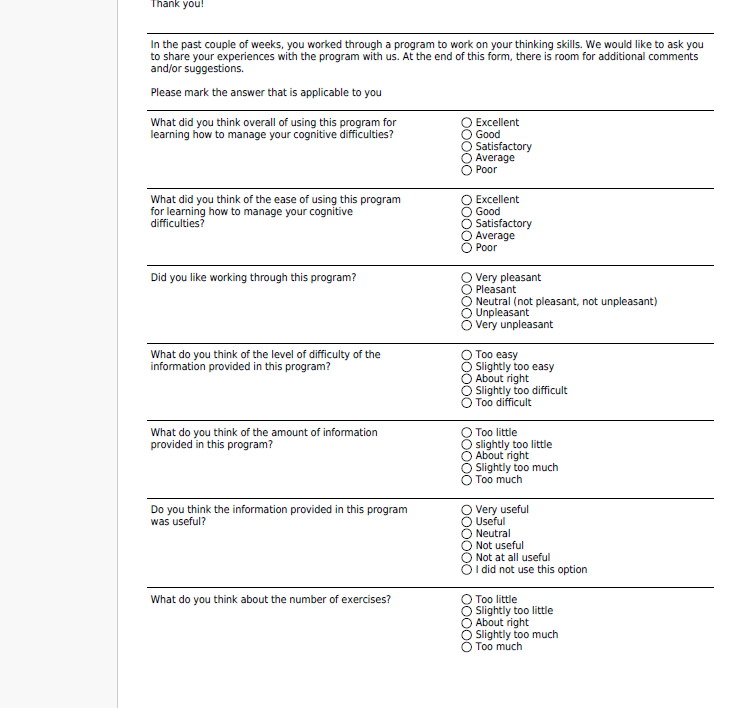
**

**
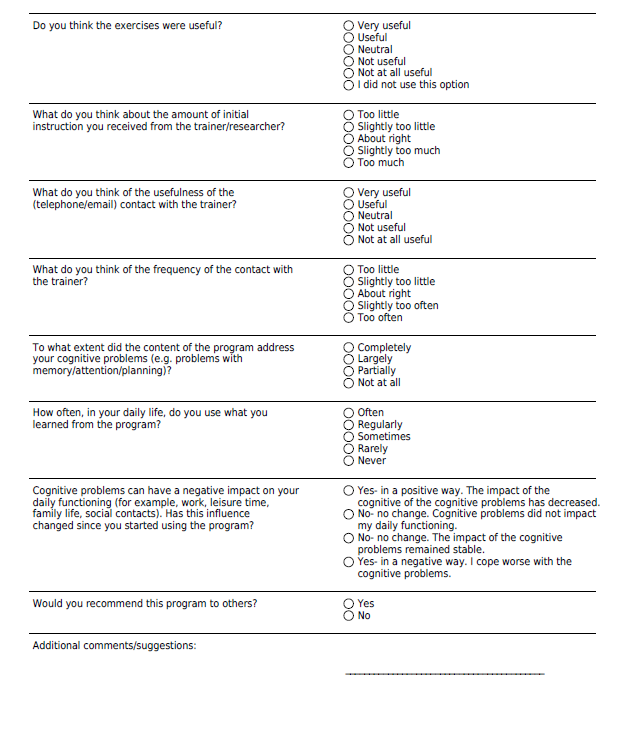
**

**Supplementary Table. 10** HealthySMS texting satisfaction survey

**
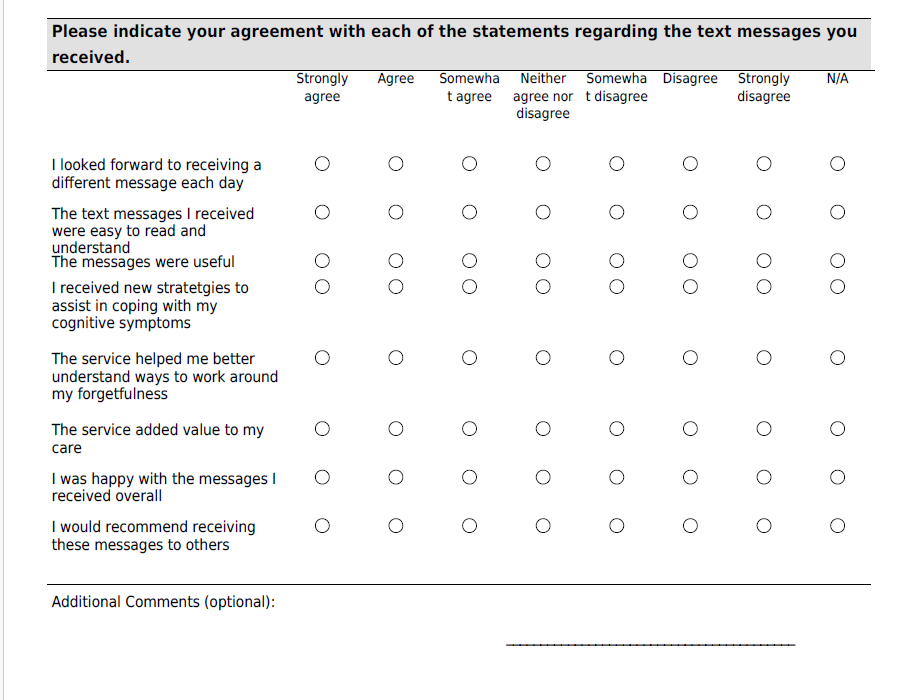
**

**SUPPLEMENTARY FIGURE CAPTIONS**

**Supplemental Figure 1**. Wechsler Adult Intelligence Scale (WAIS)-IV Working Memory Index z-score box plots at baseline and post-intervention for the GMT group.
